# Zerumbone Combined with Hydrolytic Enzymes: A Promising Strategy to Control Mixed Biofilms of *Streptococcus mutans* and Fluconazole-Resistant *Candida albicans*

**DOI:** 10.1021/acsomega.6c03152

**Published:** 2026-08-19

**Authors:** Analú Barros de Oliveira, Beatriz H. D. Panariello, Paula Aboud Barbugli, Túlio Morandin Ferrisse, Ana Claudia Pavarina

**Affiliations:** † Department of Dental Materials and Prosthodontics, School of Dentistry, 28108 São Paulo State University (UNESP), Araraquara, São Paulo 14801-903, Brazil; ‡ LECOM School of Dental Medicine, 4800 Lakewood Ranch Blvd., Bradenton, Florida 34211, United States; § Department of Diagnosis and Surgery, School of Dentistry, São Paulo State University (UNESP), Araraquara, São Paulo 14801-903, Brazil

## Abstract

This in vitro study evaluated the efficacy of Zerumbone (ZER), a sesquiterpene phytochemical extracted from the rhizomes of *Zingiber zerumbet* (L.) Smith with reported antimicrobial properties, alone or combined with Dextranase (DEX) and DNase I (DNase), against polymicrobial biofilms of fluconazole-susceptible and fluconazole-resistant *Candida albicans* and *Streptococcus mutans*. Mixed-species biofilms were formed with *C. albicans* ATCC 90028 (CaS), *C. albicans* ATCC 96901 (CaR), and *S. mutans* UA159 and assessed in inhibition and disruption assays. Treatments included ZER, DEX, and DNase, individually or in combination. Efficacy was analyzed by total biofilm mass, metabolic activity (CFU/mm^2^), and live/dead cell assessment using CLSM. Data were analyzed by one- and two-way ANOVA followed by Tukey’s posthoc test (α = 0.05). In the biofilm inhibition assay, ZER and its combinations, particularly ZER + DEX, demonstrated enhanced activity against both *C. albicans* and *S. mutans* strains. For CaS + Sm biofilms, ZER, ZER + DEX, and DNase + DEX achieved similar and the greatest reduction in CaS viability (>2.7 log_10_), whereas, for *S. mutans*, DNase + DEX, ZER, and ZER + DEX were the most effective (>2.1 log_10_). ZER + DEX also significantly reduced total microbial load in CaR + Sm biofilm. Notably, ZER + Dextranase was the only treatment that significantly reduced dry weight in susceptible biofilms and produced the highest biomass reduction in both susceptible and resistant biofilms as measured by crystal violet staining. In the biofilm disruption assay, ZER + DNase caused the greatest reduction in CaS viability (2.08 log_10_), whereas ZER and ZER + DNase were most effective against *S. mutans* (>2.5 log_10_). For CaR + Sm biofilms, ZER + DEX and ZER + DNase were among the most effective treatments. The confocal microscopy results corroborated these findings, showing that ZER and all treatments combined with ZER significantly increased the proportion of dead cells under both tested conditions. Within the limitations of this in vitro study, ZER alone or in combination with matrix-degrading enzymes (DEX and DNase), showed enhanced activity against both inhibition and disruption of mixed-species biofilms of *C. albicans* and *S. mutans*, including fluconazole-resistant strains, suggesting potential for further investigation as an adjunctive antimicrobial approach.

## Graphical abstract

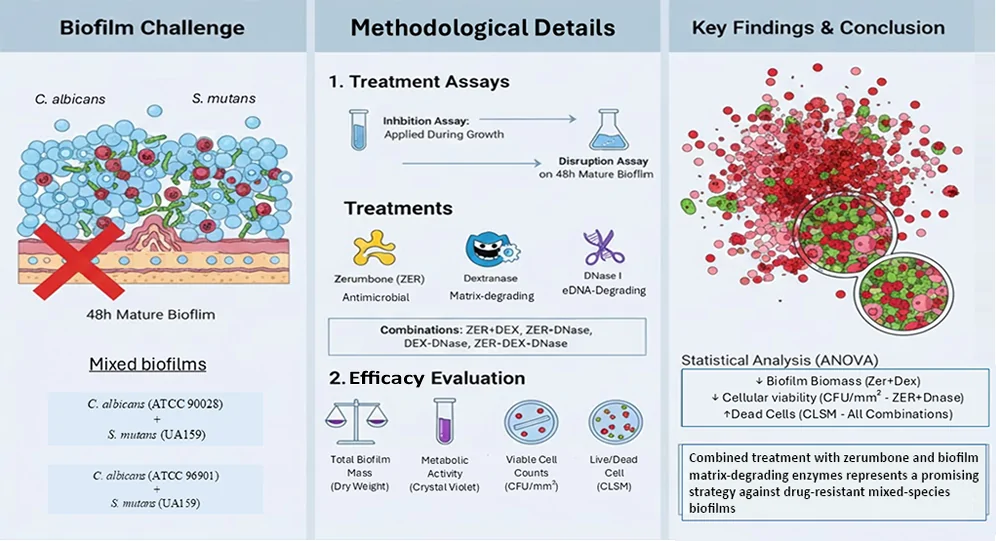

## Introduction

Oral biofilm formation results from interactions between micro-organisms, the host, and dietary factors and plays a key role in oral diseases such as caries, periodontitis, and endodontic infections. A major clinical challenge is effective disruption of the biofilm structure.^1^ Fungi are important components of oral biofilm-related infections, with *Candida albicans* being the most prevalent opportunistic species found in the oral cavity of healthy individuals (60%–70%).^2^

Under specific conditions, *C. albicans* may exhibit pathogenic behavior, resulting in oral candidiasis—a recurrent fungal infection that affects the mucosal surfaces, with approximately 95% of the cases related to *C. albicans*.^3^ Factors such as dental prostheses, reduced saliva flow, and interactions with other micro-organisms contribute to the fungal colonization and development of infection.^4^ Current international guidelines, including those developed by the European Confederation for Medical Mycology (ECMM) and the International Society of Human and Animal Mycology (ISHAM), recommend fluconazole as the first-line therapy for oropharyngeal candidiasis, particularly in immunocompromised patients, with topical agents such as nystatin or miconazole reserved for mild cases. For systemic candidiasis, including candidemia and invasive disease, echinocandins are strongly recommended as initial therapy because of their broad spectrum and fungicidal activity, while liposomal amphotericin B and azoles, such as fluconazole, serve as alternative treatments, depending on susceptibility and patient-related factors.^5^

Widespread use of fluconazole has contributed to increasing resistance in *Candida* species, representing a significant clinical concern. This resistance not only compromises treatment efficacy but also increases costs and morbidity, directly impacting the management of high-risk patients, such as those with HIV and other immunosuppressive conditions.^6^ This issue is exacerbated by polymicrobial biofilms, which enhance pathogenicity and treatment failure. In this scenario, *Streptococcus mutans*, which plays a key role in dental caries through acid production and enamel demineralization,^7^ has been shown to interact with *C*. *albicans,* especially in cases of severe early childhood caries (ECC), enhancing *S. mutans*’ cariogenic potential by promoting acid production and biofilm formation and acting as a cofactor that modulates *S. mutans*’ virulence.^8^ In addition, *S. mutans* play a significant role in the development of oral candidiasis by interacting synergistically with *C. albicans*, enhancing their hyphal formation, promoting the production of a robust extracellular matrix, acidifying the local environment through carbohydrate fermentation, and contributing to the formation of resilient polymicrobial biofilms, particularly on prosthetic surfaces.^9,10^

Increasing resistance of polymicrobial biofilms to conventional therapies is attributed to the extracellular matrix (ECM), a complex, self-produced network of polysaccharides, proteins, and extracellular DNA that surrounds microbial cells. The ECM acts as a protective barrier that limits antimicrobial penetration and contributes to treatment failure, highlighting the need for strategies that disrupt its structure.^11^ In this context, enzymatic therapies have emerged as a promising strategy.^12–15^ Enzymes such as Dextranase have shown potential to disrupt *Streptococcus spp*. biofilms in animal models by targeting ECM polysaccharides.^15^ The combined use of Dextranase and mutanase also showed an additive effect in reducing biofilm formation.^14^ Similarly, DNase targets eDNA in the ECM and has been effective in preventing *Streptococcus* biofilm formation in vitro and reducing early dental biofilms.^13^ DNase I also disrupts the extracellular matrix of *C. albicans* biofilms, improving photosensitizer penetration and enhancing efficacy of antifungal photodynamic therapy.^12^ Together, these findings suggest that biofilm removal may be improved by combining enzymes that target distinct ECM components.

In response to the urgent need for new antimicrobial strategies, natural compounds have gained increasing relevance. Zerumbone (ZER), a monocyclic sesquiterpene isolated from *Zingiber zerumbet* (L.) Smith, is a promising candidate due to its anti-inflammatory, antitumor, immunomodulatory, and antimicrobial properties.^16^ ZER disrupts the cell membrane of pathogens such as *C. albicans* and *S. mutans*, causing depolarization and increased permeability.^17^ Importantly, it also disorganizes the extracellular matrix (ECM) of *C. albicans* biofilms, including fluconazole-resistant strains.^18^ ZER reduces biomass, protein content, water-soluble and alkali-soluble polysaccharides, and extracellular DNA, which are essential for biofilm integrity and resistance.^18^ It has also inhibited mixed-species biofilms of *C. albicans* and *S. mutans* on acrylic resin surfaces.^19^ In addition to disrupting biofilm extracellular matrix and showing low cytotoxicity toward mammalian cells,^20^ ZER induces reactive oxygen species (ROS) production in cancer cells, thereby promoting oxidative stress and apoptosis.^21^

Although ZER has demonstrated promising antimicrobial activity, including disruption of biofilm structure and extracellular matrix components in both single and mixed-species biofilms, its effects have not been directly compared with enzymatic strategies targeting specific ECM components. Similarly, hydrolytic enzymes such as Dextranase and DNase I have shown efficacy in disrupting polysaccharide and extracellular DNA components of biofilms, but their performance when combined with natural compounds like ZER remains unexplored. Importantly, no study has evaluated whether combining ZER with Dextranase and DNase yields additive or synergistic effects against polymicrobial biofilms composed of both fluconazole-susceptible and fluconazole-resistant *C. albicans* and *S. mutans*. This represents a critical gap in current knowledge, particularly given the clinical relevance of polymicrobial biofilms and antifungal resistance. Therefore, this study addresses this gap by investigating the effects of ZER alone and combined with Dextranase and DNase on the inhibition and antimicrobial activity against interkingdom biofilms of *C. albicans* and *S. mutans*.

## METHODOLOGY

Biofilm treatments were performed in two distinct experimental conditions: (1) Biofilms were treated during biofilm growth to evaluate the effect of treatments on biofilm inhibition, and (2) after 48 h of biofilm growth to evaluate whether the treatments can disrupt the biofilm. Biofilms were treated with Zerumbone (ZER), Dextranase (DEX), or DNase I (DNase) and their combinations (ZER + DEX; ZER + DNase; DEX + DNase; ZER + DEX + DNase).

### Standardization of Treatment Solutions

ZER used in this study was obtained in its isolated form from Sigma–Aldrich (St. Louis, MO, USA; Z3902). According to the supplier’s instructions, the substance is crystal soluble in DMSO (≥10 mg/mL). Fresh solutions containing ZER at a concentration of 256 g/mL were prepared prior to each experiment by diluting an initial solution (10 mg/mL) in 1% DMSO to reach the final concentrations tested.^18^

The bovine pancreas DNase I (DNase) enzyme stock solution (AMPD1, Sigma–Aldrich, St. Louis, MO, USA) was prepared on the day of use in 0.1 M sodium acetate buffer (pH 5.5) at a concentration of 20 units/mL.^18^

The enzyme Dextranase (DEX) was provided by Sigma–Aldrich, St. Louis, MO, USA; D5884) at an initial concentration of 5000 μg/mL. Stock enzymes were aliquoted at a concentration of 0.5 units/μL, frozen at −80 °C, and only thawed once prior to experimental use.^22^

The treatments were performed either during biofilm growth (biofilm inhibition assay) or after biofilms were established (disruption of biofilm assay), using single solutions or combinations of ZER, DEX, and DNase, diluted in phosphate-citrate buffer (pH 6.0; Spectrum Chemical, New Brunswick, NJ, USA) to the final concentration. Details of the groups and the treatment performed are provided in Table 1.

**Table 1:** Summary of Experimental Groups and Treatment Conditions, Including the Concentrations of ZER, DNase, and DEX, and Their Corresponding Incubation Periods

Group	Treatment performed
Control	No treatment
Zerumbone (ZER)	ZER at 256 g/mL for 20 min^18^ ^a^
DNase I (DNase)	DNase I at 20 units/mL for 20 min^18^ ^a^
Dextranase (DEX)	DEX at 0.5 units/mL for 20 min^22^ ^a^
ZER + DNase	A mixed solution of ZER + DNase I (ZER 256 g/mL and DNase I 20 units/mL) was prepared and applied for 20 min^a^
ZER + DEX	A mixed solution of ZER + DEX (ZER 256 g/mL and DEX 0.5 units/mL) was prepared and applied for 20 min^a^
DNase + DEX	A mixed solution of DNase + DEX (DNase I, 20 units/mL; DEX, 0.5 units/mL) was prepared and applied for 20 min^a^
ZER + DNase + DEX	A mixed solution ZER + DNase I + DEX was prepared (ZER 256 g/mL, DNase I 20 units/mL, and DEX 0.5 units/mL) and applied for 20 min^a^

a These groups were evaluated under two experimental biofilm conditions: (1) Biofilm Inhibition Assay, in which treatment was applied during biofilm growth; and (2) Biofilm Disruption Assay, in which treatment was applied after biofilm maturation.

### Microorganisms’ Growth

Strains of fluconazole-susceptible *C. albicans* (ATCC 90028, CaS), fluconazole-resistant *C. albicans* (ATCC 96901, CaR), and *S. mutans* UA159 (ATCC 700610, Sm) were used in this study. Prior to experiments, each strain was reactivated from stock cultures by subculturing on Sabouraud Dextrose Agar (SDA) supplemented with chloramphenicol (5 mg/mL) for *C. albicans* and on Brain Heart Infusion (BHI) agar for *S. mutans*. Plates were incubated at 37 °C for 48 h under aerobic conditions for *C. albicans* and in a 5% CO_2_ atmosphere for *S. mutans*.

For preinoculum preparation, 5–10 colonies were individually transferred into 10 mL of tryptone yeast extract medium supplemented with 1% glucose (TYE + 1% glucose) and incubated at 37 °C. *S. mutans* was incubated in 5% CO_2_, while *C. albicans* was incubated under aerobic conditions. Both were incubated overnight to obtain actively growing cultures.^7,17^ The overnight cultures were then diluted (1:10 and 1:20) in fresh medium and further incubated until reaching the mid-logarithmic growth phase (approximately 4 h for *S. mutans* and 8 h for *C. albicans*). Cell density was monitored by measuring optical density at 600 nm for *S. mutans* and 540 nm for *C. albicans*, reaching OD values of 0.55 ± 0.08 and 0.60 ± 0.05, respectively.

Subsequently, cultures were adjusted to a final concentration of 10^7^ CFU/mL, centrifuged at 4000 × g for 5 min, and the resulting pellets were washed and resuspended in fresh medium to obtain standardized inocula for biofilm formation. For single-species assays, inocula were prepared in TYE + 1% glucose (CaS and CaR), while for *S. mutans* assays, TYE supplemented with 1% sucrose was used. All inocula were prepared at pH 7.1 prior to biofilm formation.

### Biofilm Inhibition Assay

The first experiment employed an approach in which treatment with the substances was performed during biofilm growth. For this, the mixed biofilms of *S. mutans and C. albicans* resistant or susceptible to fluconazole were established in the bottom of a 96-well plate as follows: 100 μL of the inoculum of each micro-organism was added to wells containing Tryptone Yeast Extract (TYE) culture medium (1% glucose). After 1.5 h of adhesion at 37 °C in 5% CO_2_ (adhesion phase), the adhered cells were meticulously washed with phosphate-buffered saline (PBS), and then were exposed to solutions of ZER, DEX, DNase, and their combinations (ZER + DEX; ZER + DNase; DEX + DNase; and ZER + DEX + DNase) for 20 min (Table 1). Then, the mixed biofilms were washed with phosphate-buffered saline (PBS) again, and the culture medium was replaced (fresh TYE, supplemented with 1% sucrose). TYE was subsequently changed every 8 h, alternating the supplementation between 1% glucose and 1% sucrose to promote biofilm maturation for 48 h.^23^ After 48 h, the biofilms were meticulously washed with PBS to remove nonadherent cells, and then biofilm analyses were performed using dry weight, crystal violet, and viable colony counts. In addition, analysis was performed by confocal laser microscopy.

### Disruption of Biofilm Assessment

The second experiment investigated the efficacy of ZER, DEX, DNase, and their combinations (ZER + DEX; ZER + DNase; DEX + DNase; and ZER + DEX + DNase) as a treatment for 48-h-mature biofilms. After the 48-h biofilm formation period, the culture medium was carefully removed, and the biofilms were gently washed with PBS before treatment application. Afterward, treatment was performed by adding 200 μL of treatment solution, as described in Table 1, directly onto the biofilms. The treatments remained in contact with the biofilms for 20 min under orbital agitation.^18^ After treatment, the biofilms were carefully washed with PBS to remove nonadhered cells, and the biofilm analyses were performed using dry weight, crystal violet, and viable colony counts. In addition, analysis was performed by confocal laser microscopy.

### Colony-Forming Units (CFU) Counting

To quantify viable micro-organism cells in the biofilm suspensions, serial dilutions (10^–1^ to 10^–6^) were performed. Triplicate aliquots (5 μL) of all dilutions were then plated on Petri dishes containing three different culture media: SDA supplemented with chloramphenicol for *C. albicans*, BHI supplemented with amphotericin B for *S. mutans*, and unsupplemented BHI for total microbiota. After incubation for 48 h, colony counts were conducted, and the number of colony-forming units (CFU/mm^2^) was calculated.^23^ The experiments were performed in quadruplicate on three independent occasions (*n* = 12).

### Analysis of Total Dry Weight

To quantify total biofilm mass, 0.1 mL of the biofilm suspension was pipetted onto preweighed and labeled aluminum foil. The samples were dried in an oven at 100 °C and subsequently reweighed using a precision balance. The total biofilm dry weight was determined by subtracting the initial weight of the preweighed aluminum foil and the weight of the 0.89% NaCl solution from the final weight.^24^ The experiments were performed in quadruplicate on three independent occasions (*n* = 12).

### Total Biofilm Reduction Index

The overall effect of the treatments on dual-species biofilms was assessed using a Total Biofilm Reduction Index, calculated by integrating the effects of each treatment on fungal viability, bacterial viability, and total biofilm biomass. This index was calculated based on the relative values of *C. albicans* and *S. mutans* CFU counts and biofilm dry weight (DW), using the untreated control group as the reference.

For biofilm inhibition assays, in which treatments were applied during biofilm development, the calculated index was designated as the Total Biofilm Inhibition (TBI). For biofilm disruption assays, in which treatments were applied to established 48-h mature biofilms, the same calculation was designated as the Total Biofilm Disruption (TBD). The TBI and TBD indices were calculated as follows: TBI or TBD = I fungal CFU × I bacterial CFU × I DW = [(fungal CFU treatment/fungal CFU control) × 100] × [(bacterial CFU treatment/bacterial CFU control) × 100] × [(DW treatment/DW Control) × 100].^22^ The experiments were performed in quadruplicate on three independent occasions (*n* = 12).

### Biofilms Assay by Crystal Violet Staining

After treatments, the medium was gently removed by washing three times with 200 μL of PBS. Then, 200 μL of 0.1% crystal violet was added to each well for biofilm staining. After incubating the dye for 25 min, excess dye was removed by rinsing three times with 200 μL of sterile distilled water, and the dye was immediately decolorized with 200 μL of 95% ethanol, which was left for 15 min. Biofilm quantification was performed by measuring the suspension’s optical density (OD) at 570 nm using an ELISA reader.^25^ The experiments were performed in quadruplicate on three independent occasions (*n* = 12).

### Confocal Laser Scanning Microscopy (CLSM)

Mixed biofilm cultures were cultivated in 96-well plates (SensoPlate—Greiner Bio-One Brazil). For both assays, the experimental groups were evaluated in quadruplicate on three independent occasions (*n* = 12) using confocal fluorescence microscopy (Carl Zeiss LSM 800 Airyscan with GaAsp detector, Germany). After biofilm formation, 200 μL of 0.89% NaCl was used to wash the wells, and then 100 μL of a 1:1000 dilution of SYTO 9 and Propidium Iodide (PI) solution in 1× PBS from the LIVE/DEAD BacLight kit (Invitrogen, Carlsbad, CA, USA) was added. SYTO 9 is a green-fluorescent, cell-permeable nucleic acid stain that readily passes through the membranes of both prokaryotic (bacteria) and eukaryotic­(fungi) cells, while PI marks dead cells (red).^22^ The plates were incubated for 30 min. Afterward, the wells were washed again with 100 μL of 0.89% NaCl. For image acquisition, at 10× magnification, 100 μL of 0.89% NaCl was added to the well plates. The configuration settings were as follows: SYTO 9 - excitation: 488 nm; detection: 520 nm/propidium iodide: excitation: 561 nm; detection: 620 nm). Image analysis was performed using Zen Blue LITE software (version 2.3). The fluorescence of each CLSM channel (Live/Dead) was quantified using Zen Blue LITE software, and then the proportions of live and dead micro-organisms were calculated for each group.

### Data Analysis

Data were checked for normality (Shapiro-Wilk test) and homoscedasticity (Levene’s test). For viability, dry weight, biomass, and biofilm reduction index, the Kruskal–Wallis test was performed, and Dunn’s post hoc test was applied for multiple comparisons. For CLSM quantitative results, a one-way analysis of variance (ANOVA) with Tukey’s post hoc test for multiple comparisons among groups was conducted. The significance level used was 5% (α = 0.05). Analysis was performed using IBM SPSS Statistics for Windows, version 20.0 (IBM Corp.)

## Results

### Evaluation of the Treatments on Biofilm Inhibition: Viability of Fluconazole-Susceptible and Fluconazole-Resistant *Candida albicans* with *Streptococcus mutans* in Dual-Species Biofilms

Figure 1 shows the effect of treatments on the viability of susceptible (*C. albicans* 90028) and resistant (*C. albicans* 96901) *Candida albicans* strains in dual-species biofilms with *Streptococcus mutans* during the biofilm inhibition assay. Cell viability was expressed as log_10_ CFU/mL.

**Figure 1:**
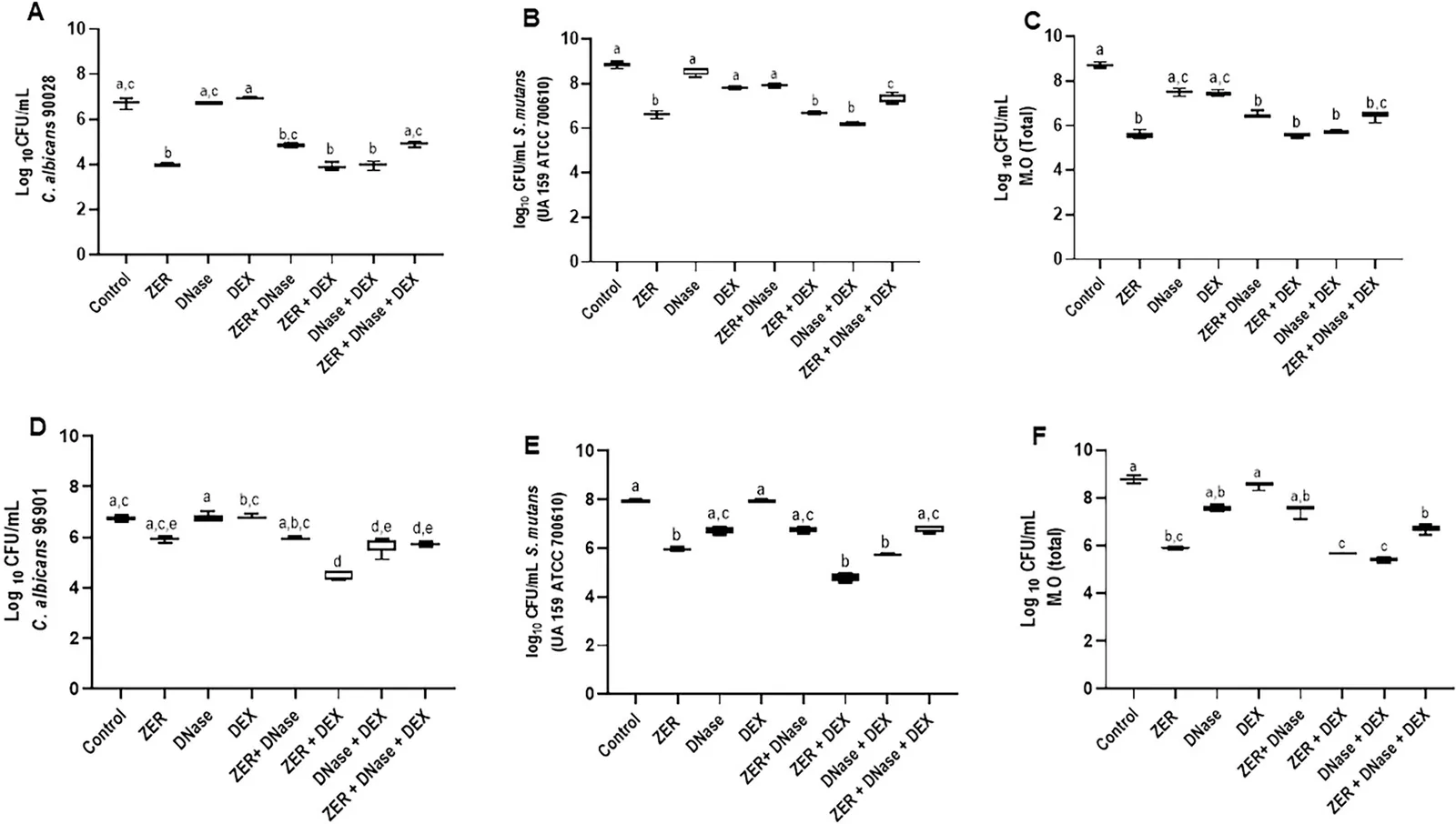
Effect of treatments on the viability of susceptible and resistant *Candida albicans* strains in dual-species biofilms with *Streptococcus mutans* during the biofilm inhibition assay. Cell viability was expressed as log_10_ CFU/mL. Panels A–C represent the susceptible dual-species biofilm model composed of *C. albicans* ATCC 90028 and *S. mutans* UA159: (A) fungal viability; (B) bacterial viability; and (C) total microorganism viability. Panels D–F represent the resistant dual-species biofilm model composed of *C. albicans* ATCC 96901 and *S. mutans* UA159: (D) fungal viability; (E) bacterial viability; and (F) total microbial viability. Data are presented as box plots showing the median, interquartile range, and minimum and maximum values. The treatment groups consisted of Control, ZER, DNase, DEX, ZER + DNase, ZER + DEX, DNase + DEX, and ZER + DNase + DEX. Statistical comparisons among groups were performed using the Kruskal–Wallis test followed by Dunn’s post hoc test for multiple comparisons. Different letters indicate statistically significant differences between groups (*p* ≤ 0.05). CFU = colony-forming units; M.O. = micro-organisms.

Compared to the control, a significant reduction in CaS (*C. albicans* 90028) viability was reached after the application of ZER + DEX (2.82 log_10_; *p* < 0.0001), ZER (2.74 log_10_; *p* < 0.0001), and DNase + DEX (2.73 log_10_; *p* < 0.0001). The differences among these treatments were not significant (*p* > 0.05). It was also noted that there were significant reductions in CaS viability for the groups ZER + DNase (1.87 log_10_; *p* < 0.0001) and ZER + DNase + DEX (1.82 log_10_; *p* < 0.001) compared to the control. The single treatments with DNase and DEX did not show significant reduction compared to the control (*p* > 0.05) (Figure 1A).

For *S. mutans*, significant reductions were observed in biofilms treated with DNase + DEX (2.70 log10 CFU/mL; *p* < 0.0001), ZER (2.19 log10 CFU/mL; *p* < 0.0001), and ZER + DEX (2.15 log10 CFU/mL; *p* < 0.0001) compared with the control. There is no statistically significant difference among the DNase + DEX, ZER, and ZER + DEX treatments (*p* > 0.05). The combination of treatments (ZER + DNase + DEX) achieved a 1.35 log_10_ reduction in viability, with a significant difference compared to the control (*p* = 0.0008). The single treatments with DNase or DEX did not show a significant difference compared to the control (*p* > 0.05), nor did the combination of ZER + DNase (*p* > 0.05) (Figure 1B).

In assessing total microbial viability of CaS + Sm biofilms, the greatest reduction was observed with ZER, which achieved a 3.15 log_10_ decrease (*p* < 0.0001). This was closely followed by ZER + DEX, with a 3.07 log_10_ reduction (*p* < 0.0001), and by DNase + DEX, which resulted in a 2.97 log_10_ reduction (*p* < 0.0001). The combination of ZER + DNase showed a 2.24 log_10_ reduction (*p* < 0.0047), as did ZER + DNase + DEX, which also achieved a 2.24 log_10_ reduction (*p* < 0.0001). All treatments were compared to the control group.

There is no significant difference among the groups ZER, ZER + DEX, ZER + DNase, DNase + DEX, and ZER + DNase + DEX (*p* > 0.05). In biofilms that received single treatments with DNase and DEX, no significant difference in microbial load reduction was observed, compared to the control (*p* > 0.05) (Figure 1C).

For CaR, significant reductions in viability were achieved after application of ZER + DEX (2.72 log_10_; *p* < 0.0001), ZER + DNase + DEX (1.00 log_10_; *p* < 0.0001), and DNase + DEX (0.93 log_10_; *p* < 0.0001) compared to the control. The single treatment with DNase (*p* > 0.05), DEX (*p* > 0.05), and the combined treatment ZER + DNase (*p* = 0.3137) did not show a significant difference in viability, compared to the control (Figure 1D).

For *S. mutans*, a significant reduction was reached in the biofilms that received ZER + DEX (2.21 log_10_; *p* < 0.0001), DNase + DEX (2.19 log_10_; *p* < 0.0001) and ZER (1.96 log_10_; *p* < 0.0001) compared to the control (Figure 1E). The differences in *S. mutans* viability among ZER + DEX, DNase + DEX and ZER were not significant (*p* > 0.05). The other treatments, such as DNase, DEX, ZER + DNase, and ZER + DNase + DEX, did not present significant reduction in *S. mutans* viability compared to the control (*p* > 0.05).

In evaluating the total microbial viability of *CaR+Sm* biofilms, we observed significant reductions with the following treatments compared to the control group: ZER + DEX showed a reduction of 3.11 log_10_ CFU/mL (*p* < 0.0001), DNase + DEX resulted in 3.35 log_10_ CFU/mL (*p* < 0.0001), ZER alone led to 2.87 log_10_ CFU/mL (*p* < 0.0001), DNase alone resulted in 1.22 log_10_ CFU/mL (*p* < 0.0001), and ZER + DNase showed a reduction of 1.05 log_10_ CFU/mL (*p* < 0.0001). These results are illustrated in Figure 1F. The DEX treatment did not show a significant difference, compared with the control (*p* > 0.05).

### Evaluation of the Treatments on Biofilm Inhibition: Dry Weight, Total Biofilm Inhibition (TBI), and Total Biomass

Figure 2 shows the effects of treatments on biofilm dry weight, total biofilm inhibition, and total biomass in dual-species biofilms formed by fluconazole-susceptible or fluconazole-resistant *Candida albicans* strains with *Streptococcus mutans*.

**Figure 2:**
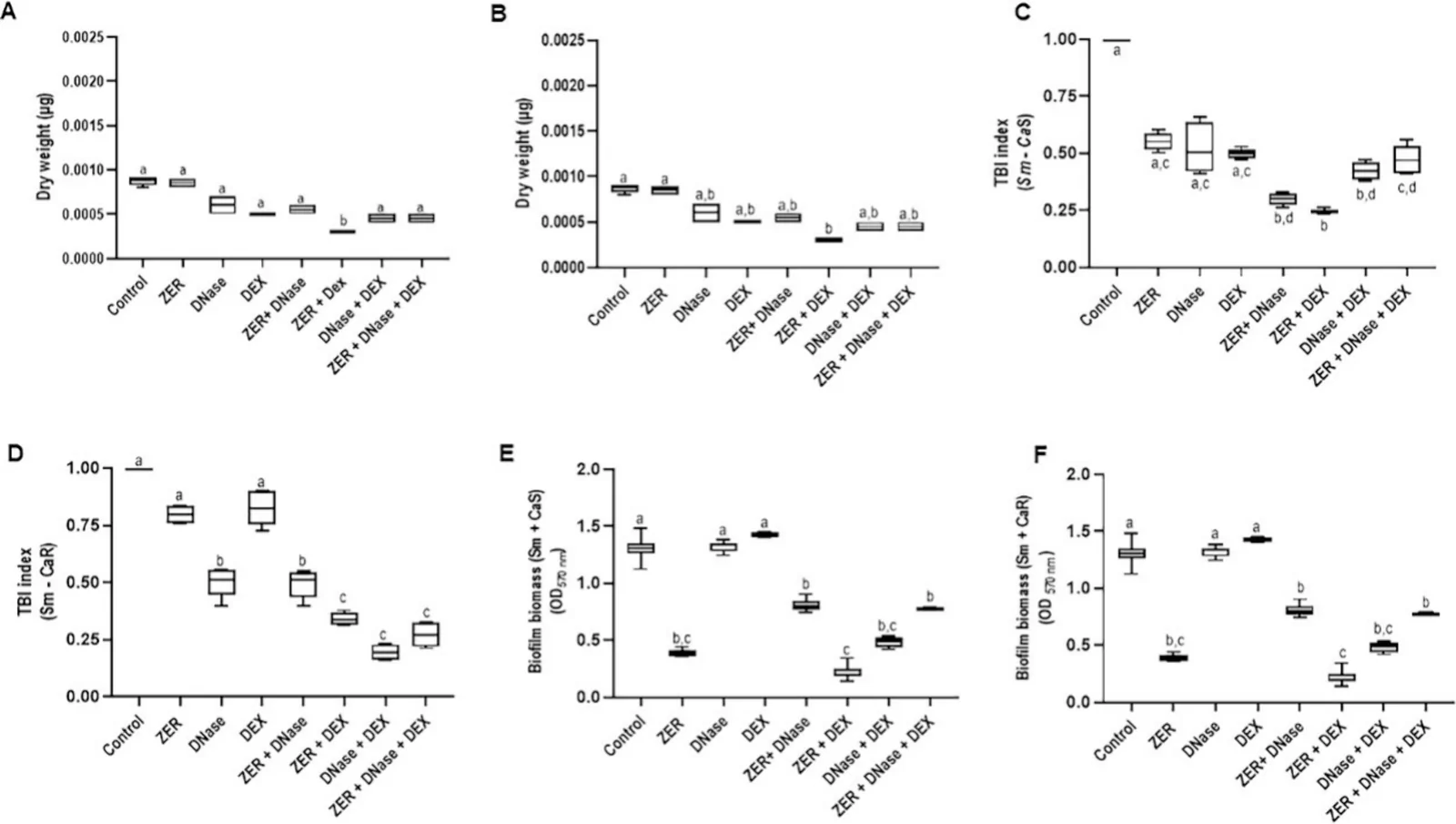
Effect of treatments on biofilm dry weight (μg), Total Biofilm Inhibition (TBI), and total biomass in dual-species biofilm inhibition assays formed with fluconazole-susceptible or fluconazole-resistant *Candida albicans*. Panels (A), (C), and (E) show results for the susceptible model, and panels (B), (D), and (F) show results for the resistant model. Dual-species biofilms were composed of *Streptococcus mutans* UA159 with either fluconazole-susceptible or fluconazole-resistant *C. albicans*. Data are presented as box plots showing the median, interquartile range, and minimum and maximum values. Treatment groups included Control, ZER, DNase, DEX, ZER + DNase, ZER + DEX, DNase + DEX, and ZER + DNase + DEX. Statistical comparisons were performed using the Kruskal–Wallis test, followed by Dunn’s post hoc test. Different letters indicate significant differences between groups (*p* < 0.05). TBI = Total Biofilm Inhibition; OD = optical density; CaS = fluconazole-susceptible *C. albicans*; CaR = fluconazole-resistant *C. albicans*.

ZER + DEX was the only treatment that reduced the dry weight in CaS + Sm biofilms, compared to the control (*p* < 0.001) (Figure 2A). The other groups did not show a statistically significant difference compared to the control (*p* > 0.05). The same results were obtained for dry weight assay in resistant dual-species biofilm (CaR + Sm) (Figure 2B).

Regarding the total biofilm index (TBI) (Figure 2C), for CaS + Sm biofilms, the lowest TBI was achieved with ZER + DEX (*p* < 0.0001), followed by DNase + DEX (*p* < 0.0001) and ZER + DNase (*p* < 0.0001), all of which showed significant differences compared to the control. The differences between ZER + DEX, DNase + DEX, and ZER + DNase, and DEX were not significant (*p* > 0.05). In addition, no significant reduction in TBI was observed in biofilms treated with ZER (*p* > 0.05), DNase (*p* < 0.05), and DEX (*p* > 0.05), compared to the control.

Apart from single treatment with DEX and ZER, all other treatments applied to CaR + Sm biofilms showed significantly lower TBI than the control group (*p* < 0.0001). However, the greatest reduction was observed for DNase + DEX, followed by ZER + DEX and ZER + DNase + DEX (Figure 2D). Moreover, a significant reduction was also observed for biofilms treated with ZER + DNase and DNase groups compared to the control (*p* < 0.0001). No significant differences were found for single treatment with ZER or DEX, compared with the control group (*p* > 0.05).

Regarding total biomass evaluated by the crystal violet assay, the best biomass reduction for both biofilms evaluated (CaS+Sm and CaR+Sm) was observed with the ZER + DEX group, followed by ZER, DNase + DEX, and ZER + DNase + DEX combinations (Figure 2E and 2F). These treatments were significantly different than the control (*p* < 0.0001). On the other hand, the treatments with DNase and DEX did not show statistically significant differences compared to the control (*p* > 0.05).

### Evaluation of the Treatments on Biofilm Disruption: Viability of Fluconazole-Susceptible and Fluconazole-Resistant *Candida albicans* with *Streptococcus mutans* in Dual-Species Biofilms

Figure 3 shows the effect of treatments on the viability of susceptible (*C. albicans* 90028) and resistant (*C. albicans* 96901) *Candida albicans* strains in dual-species biofilms with *Streptococcus mutans* during the biofilm disruption assay. Cell viability was expressed as log_10_ CFU/mL.

**Figure 3:**
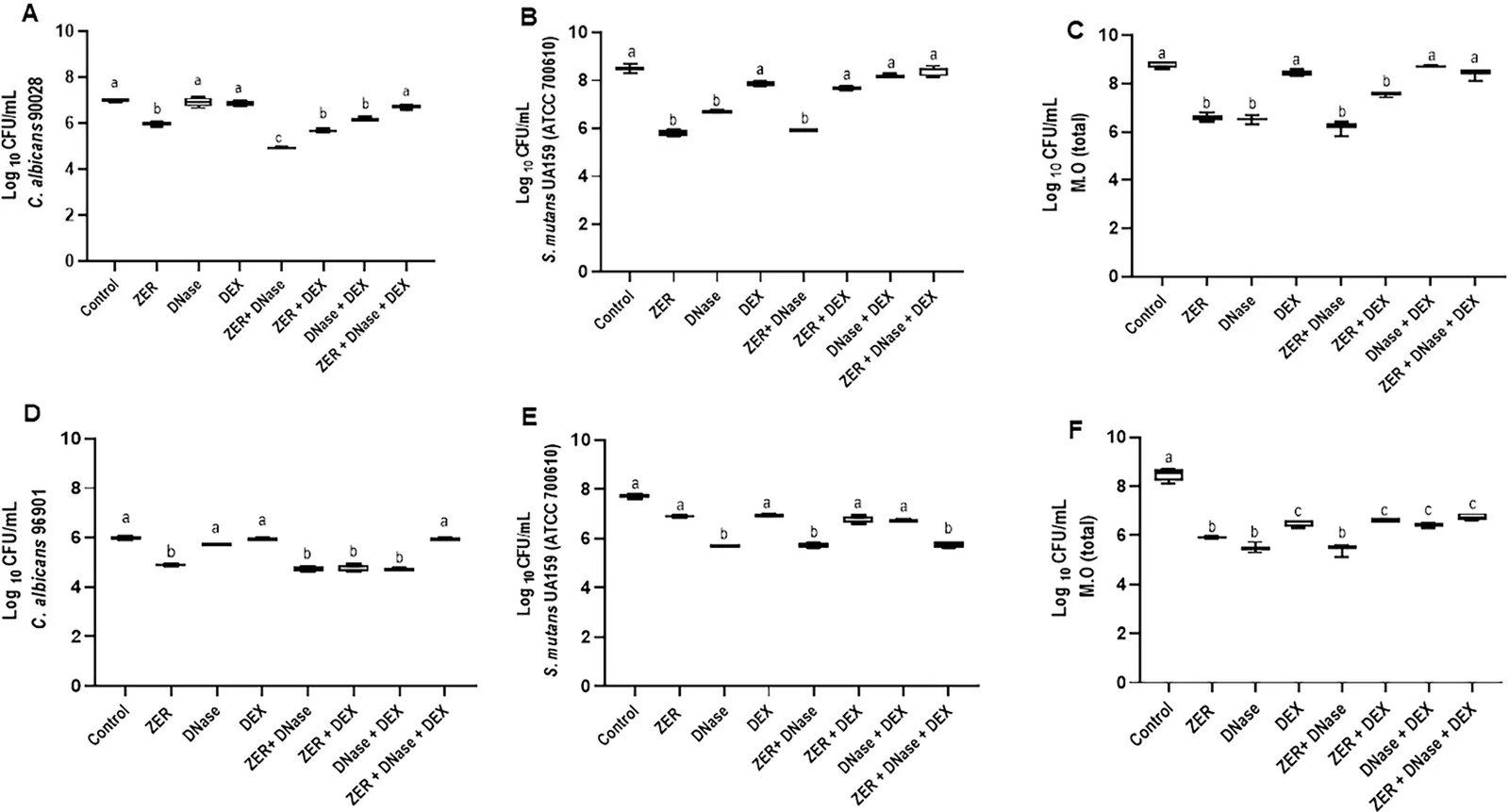
Effects of treatments on cell viability in fluconazole-susceptible and fluconazole-resistant *Candida albicans* dual-species biofilms with *Streptococcus mutans* during the biofilm disruption assay. Cell viability was expressed as log10 CFU/mL following treatment of preformed dual-species biofilms composed of *C. albicans* and *S. mutans*. Panels A–C represent the fluconazole-susceptible dual-species biofilm model composed of *C. albicans* ATCC 90028 and *S. mutans* UA159: (A) fungal viability; (B) bacterial viability; and (C) total microbial viability. Panels D–F represent the fluconazole-resistant dual-species biofilm model composed of *C. albicans* ATCC 96901 and *S. mutans* UA159: (D) fungal viability; (E) bacterial viability; and (F) total microbial viability. Data are presented as box plots showing the median, interquartile range, and minimum and maximum values. Treatment groups included Control, ZER, DNase, DEX, ZER + DNase, ZER + DEX, DNase + DEX, and ZER + DNase + DEX. Statistical comparisons among groups were performed using the Kruskal–Wallis test followed by Dunn’s post hoc test for multiple comparisons. Different letters indicate statistically significant differences between groups (*p* < 0.05). CFU = colony-forming units; M.O. = microorganisms.

Significant reduction in CaS viability was reached after the application of Zer + DNase (2.08 log_10_; *p* < 0.0001) treatment followed by ZER+DEX (1.33 log_10_; *p* < 0.0001), ZER (1.01 log_10_; *p* < 0.0001) and DNase + DEX (0.84 log_10_; *p* < 0.0001) compared to the control (Figure 3A). However, DNase, DEX and the association of ZER + DNase + DEX did not show significant difference compared to the control (*p* > 0.05).

A significant reduction in *S. mutans* was observed in the biofilms that received ZER (2.70 log_10_; *p* < 0.0001) and ZER + DNase (2.58 log_10_; *p* < 0.0001), followed by DNase (1.81 log_10_; *p* < 0.0001), compared with the control (Figure 3B). No significant reduction in viability was observed in the DEX, ZER + DEX, DNase + DEX, and ZER + DNase + DEX groups compared with the control (*p* > 0.05).

The results of total microbial viability (CaS + Sm) showed significant reduction after ZER + DNase (2.43 log_10_; *p* < 0.0001), ZER (2.20 log_10_; *p* < 0.0001), DNase (2.20 log_10_; *p* < 0.0001) and ZER + DEX groups (1.12 log_10_; *p* < 0.01) when compared to the control group. (Figure 3C). In addition, the other treatments (DEX, DNase + DEX, and ZER + DNase + DEX) did not show significant differences, compared to the control (*p* > 0.05).

Significant reduction in CaR viability was achieved after application of DNase + DEX (1.28 log_10_; *p* < 0.0001), ZER + DNase (1.23 log_10_; *p* < 0.0001), ZER + DEX (1.20 log_10_; *p* < 0.0001) and ZER (1.07 log_10_; *p* < 0.01) comparing them to the control. The differences among these treatments were not significant (*p* > 0.05). The groups ZER + DNase + DEX, DNase, and DEX did not show significant differences compared to the control (*p* > 0.05) (Figure 3D).

Significant reduction in *S. mutans* was observed in the biofilms that received DNase (2.02 log_10_; *p* < 0.0001), ZER + DNase + DEX (1.98 log_10_; *p* < 0.0001), and ZER + DNase (1.96 log_10_; *p* < 0.0001) treatment comparing them to the control. Moreover, the differences among these groups were not considered significant (*p* > 0.05). The other treatments, such as ZER, DEX, ZER + DEX and DNase + DEX did not show significant differences in comparison with the control (*p* > 0.05) (Figure 3E).

For total microbial viability, significant reductions were observed for DNase (3.14 log_10_; *p* < 0.0001), ZER + DNase (3.04 log_10_; *p* < 0.0001), and ZER (2.64 log_10_; *p* < 0.0001) treatments compared to the control. However, no significant differences were noted among the DNase, ZER + DNase, and ZER treatments (*p* > 0.05) (Figure 3F).

### Evaluation of the Treatments on Biofilm Disruption: Dry Weight, Total Biofilm Disruption (TBD), and Total Biomass

Figure 4 shows the effects of treatments on biofilm dry weight, TBD, and total biomass in preformed dual-species biofilms containing fluconazole-susceptible and fluconazole-resistant strains. The dry weight (μg) results for CaS + Sm biofilms demonstrated that ZER + DEX (*p* < 0.0001) promoted the most significant reductions (*p* < 0.01) compared to the control group. The other groups did not show significant reductions in dry weight compared to the control group (all *p* > 0.05) (Figure 4A). For the CaR + Sm biofilm, the greatest reductions in dry weight were achieved with the combination of DNase and DEX (*p* < 0.0001) as well as ZER, DNase, and DEX (*p* < 0.0001) when compared to the control group. Additionally, treatments with DNase, ZER + DEX, ZER + DNase, and DEX also demonstrated statistically significant reductions (all *p* < 0.05), though these were not as significant as those seen with DNase + DEX and ZER + DNase + DEX (see Figure 4B). On the other hand, ZER treatment did not show a significant difference compared to the control (*p* > 0.05) (Figure 4A and 4B).

**Figure 4:**
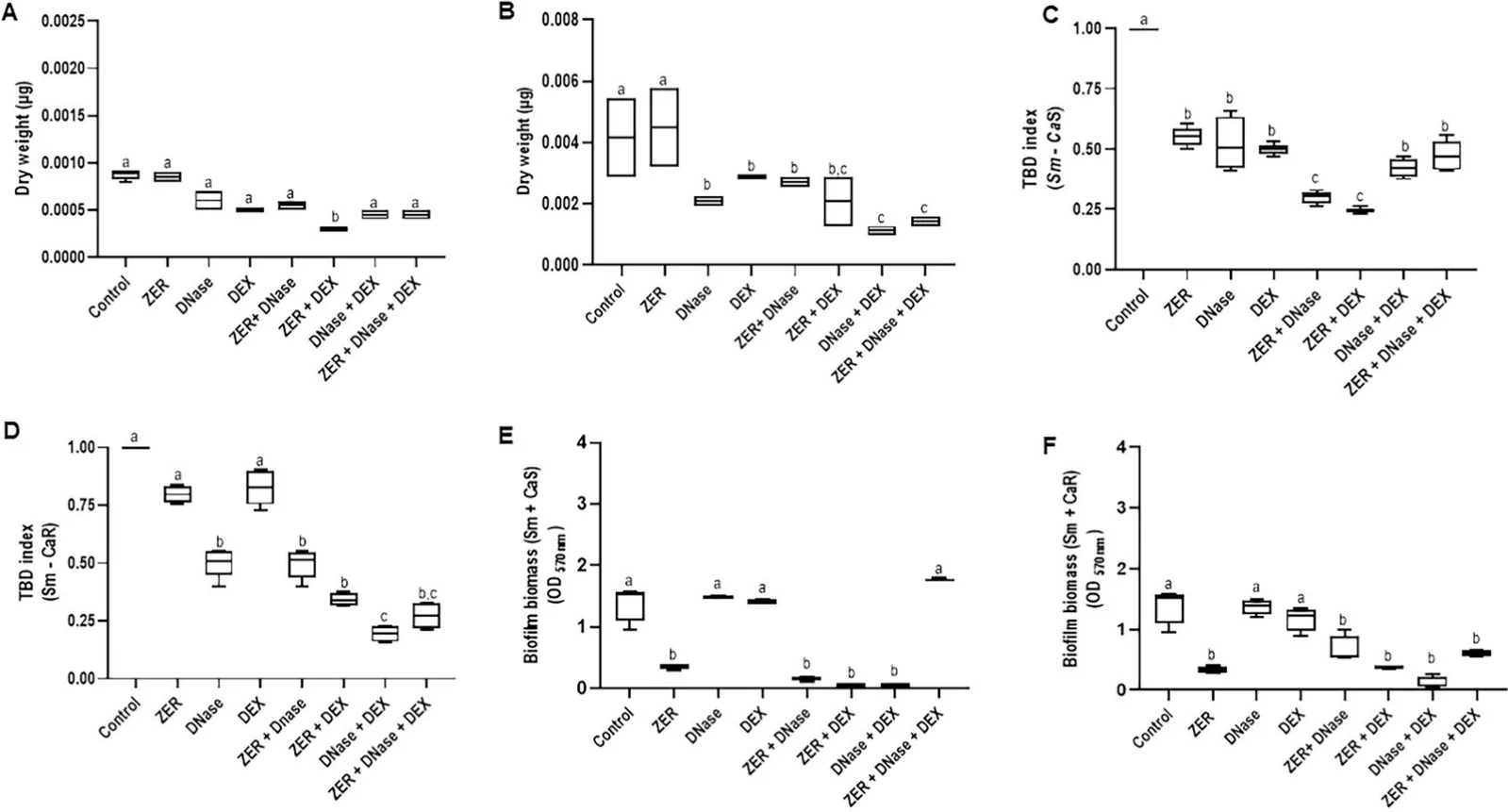
Effects of treatments on biofilm dry weight, Total Biofilm Disruption (TBD), and total biomass in preformed fluconazole-susceptible and fluconazole-resistant dual-species biofilms. Panels (A) and (B) show the dry weight of biofilms in the fluconazole-susceptible and fluconazole-resistant dual-species biofilm models, respectively. Panels (C) and (D) show the TBD index in the fluconazole-susceptible and fluconazole-resistant dual-species biofilm models, respectively. Panels (E) and (F) show total biomass in the fluconazole-susceptible and fluconazole-resistant dual-species biofilm models, respectively. The fluconazole-susceptible dual-species biofilm model consisted of *S. mutans* UA159 and fluconazole-susceptible *C. albicans*, whereas the fluconazole-resistant dual-species biofilm model consisted of *S. mutans* UA159 and fluconazole-resistant *C. albicans*. Data are presented as box plots showing the median, interquartile range, and minimum and maximum values. Treatment groups included Control, ZER, DNase, DEX, ZER + DNase, ZER + DEX, DNase + DEX, and ZER + DNase + DEX. Statistical comparisons among groups were performed using the Kruskal–Wallis test followed by Dunn’s post hoc test for multiple comparisons. Different letters indicate statistically significant differences between groups (*p* < 0.05). TBD = Total Biofilm Disruption; OD = optical density; Sm = *S. mutans*; CaS = fluconazole-susceptible *C. albicans*; CaR = fluconazole-resistant *C. albicans*.

A total biofilm disruption (TBD) index was calculated using viability and dry weight values.^22^ For CaS + Sm biofilms, ZER + DEX (*p* < 0.0001) and ZER + DNase (*p* < 0.0001) demonstrated the greatest reduction in TBD (see Figure 4C). For CaR + Sm biofilms, the combinations of DNase + DEX (*p* < 0.0001) and ZER + DNase + DEX (*p* < 0.0001) also yielded significant reductions in TBD results. This was followed by ZER + DEX (*p* < 0.0001), DNase (*p* < 0.01), and ZER + DNase (*p* < 0.01) as shown in Figure 4D. Notably, the treatments with ZER and DEX did not show significant differences when compared to the control (*p* > 0.05).

When assessing biomass in CaS + Sm biofilms via the crystal violet assay, the most significant reductions were observed for ZER + DEX (*p* < 0.0001), DNase + DEX (*p* < 0.0001), ZER + DNase (*p* < 0.01), and ZER (*p* < 0.0001). All these results were compared to the control. The other groups did not show significant differences compared to the control (*p* > 0.05) (Figure 4E). For CaR + Sm biofilms, the most significant biomass reduction was observed with DNase + DEX (*p* < 0.0001), ZER + DEX (*p* < 0.0001), ZER (*p* < 0.0001), and ZER + DNase + DEX (*p* < 0.001) compared to the control. The DNase and DEX groups did not show significant differences compared to the control group (*p* > 0.05) (Figure 4F).

### Confocal Laser Scanning Microscopy (CLSM)

The cell viability findings following treatment of dual-species biofilms were corroborated by confocal laser scanning microscopy (CLSM), as shown in Figure 5 for the biofilm inhibition assay and Figure 6 for the biofilm disruption assay. In the biofilm inhibition assay (Figure 5), the quantification of live/dead cells in CLSM highlighted a significant reduction in cell viability in susceptible and resistant biofilms treated with ZER alone and all combined treatments (ZER + DNase, ZER + DEX, DNase + DEX and ZER + DNase + DEX), when compared to the control group (*p* < 0.001).

For the biofilms formed with the susceptible *C. albicans* strain (Figure 5A and 5B), ZER alone and the combination of all treatments reached approximately the same live/dead cell proportions (∼30%/70%). Among these treatments, no significant differences were observed (*p* > 0.05). For the biofilms formed with the resistant *C. albicans* strain, the highest reduction in cell viability was reached with ZER + DEX and DNase + DEX (∼37%/ 63%), followed by ZER + DNase + DEX (∼47%/53%) (Figure 5C and 5D). All these treatments were significantly different from the control group (*p* < 0.001), and ZER + DEX and DNase + DEX were significantly different from ZER, DNase and ZER + DNase + DEX (*p* < 0.01).

**Figure 5:**
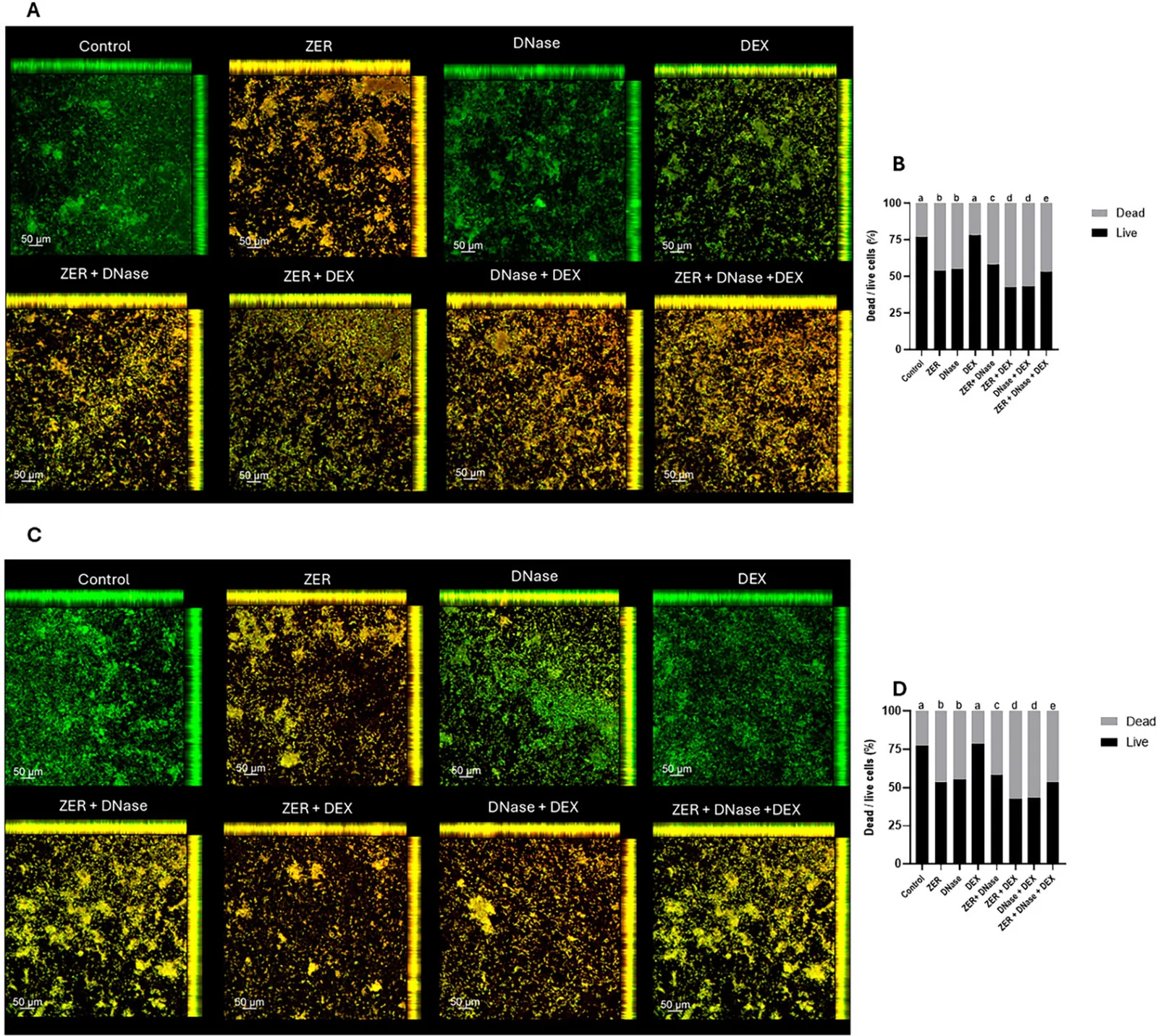
CLSM analysis of live and dead cells in fluconazole-susceptible and fluconazole-resistant dual-species biofilms during the biofilm inhibition assay. Panels (A) and (B) show representative qualitative CLSM images of dual-species biofilms after treatment, with live cells stained in green and dead cells stained in yellow. Panel (A) represents the fluconazole-susceptible dual-species biofilm model, whereas panel (C) represents the fluconazole-resistant dual-species biofilm model. Panels (B) and (D) show the quantitative analysis of live and dead cells in the fluconazole-susceptible and fluconazole-resistant dual-species biofilms, respectively. The fluconazole-susceptible dual-species biofilm model consisted of *S. mutans* UA159 and fluconazole-susceptible *C. albicans*, whereas the fluconazole-resistant dual-species biofilm model consisted of *S. mutans* UA159 and fluconazole-resistant *C. albicans*. The treatment groups consisted of Control, ZER, DNase, DEX, ZER + DNase, ZER + DEX, DNase + DEX, and ZER + DNase + DEX. Statistical comparisons among groups were performed using one-way ANOVA followed by Tukey’s post hoc test for multiple comparisons. Different letters indicate statistically significant differences between groups (*p* < 0.05). CLSM = confocal laser scanning microscopy.

In the biofilm disruption assay (Figure 6), quantification of live/dead cells by CLSM revealed a significant reduction in cell viability in susceptible and resistant biofilms compared to the control group (*p* < 0.001). For susceptible biofilms (Figure 6A and 6B), the highest reduction in the proportion of live/dead cells was achieved with ZER + DNase treatment (∼35%/65%), followed by ZER and DNase alone, ZER + DEX, and ZER + DNase + DEX (55%/45%). The differences observed with ZER + DNase treatment were significant compared with ZER and DNase alone, ZER + DEX, and ZER + DNase + DEX (*p* < 0.001).

**Figure 6:**
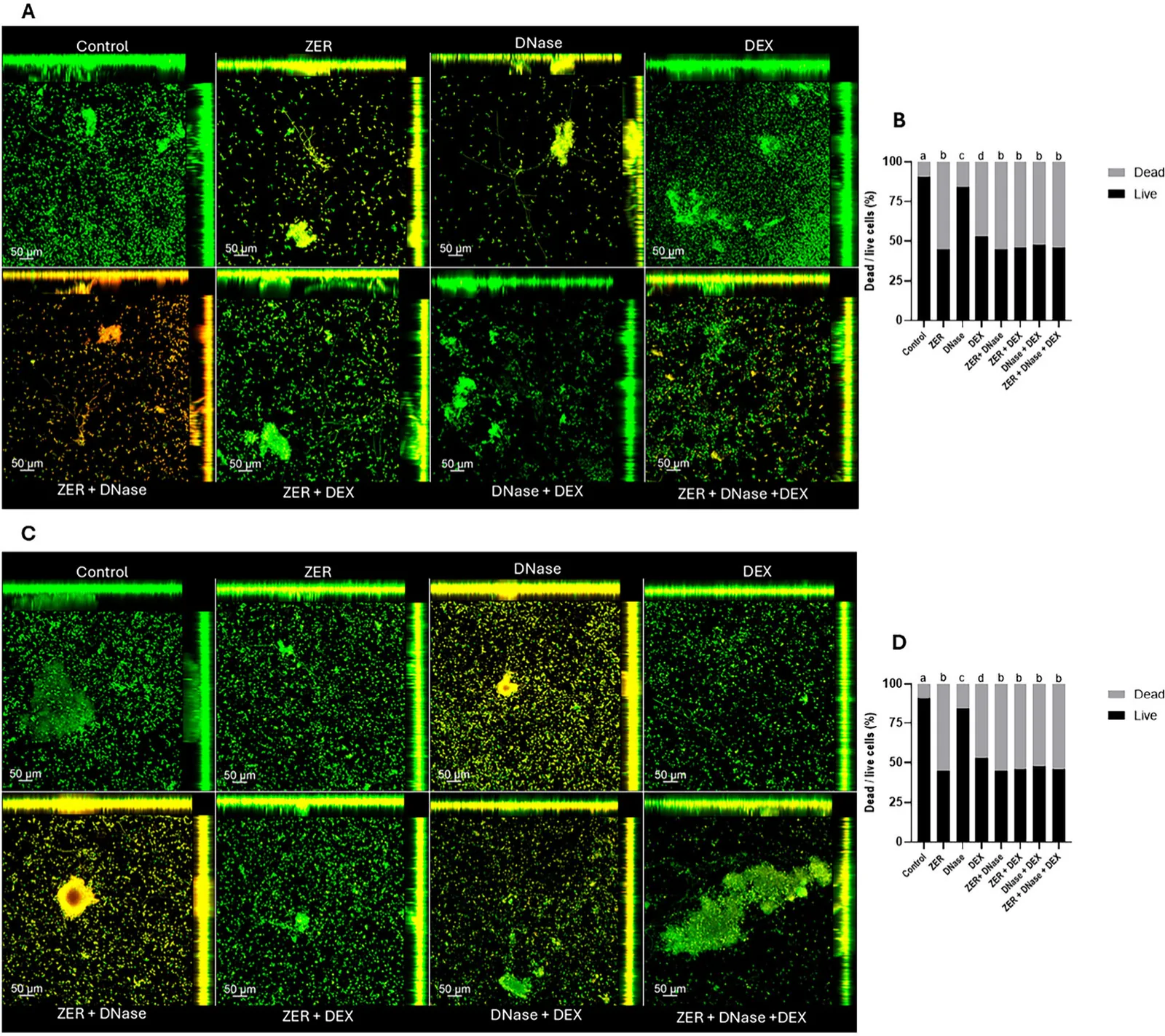
CLSM analysis of live and dead cells in preformed fluconazole-susceptible and fluconazole-resistant dual-species biofilms during the biofilm disruption assay. Panels (A) and (B) show representative qualitative CLSM images of dual-species biofilms after treatment, with live cells stained in green and dead cells stained in yellow. Panel (A) represents the fluconazole-susceptible dual-species biofilm model, whereas panel (B) represents the fluconazole-resistant dual-species biofilm model. Panels (C) and (D) show the quantitative analysis of live and dead cells in the fluconazole-susceptible and fluconazole-resistant dual-species biofilms, respectively. The fluconazole-susceptible dual-species biofilm model was composed of *S. mutans* UA159 and fluconazole-susceptible *C. albicans*, whereas the fluconazole-resistant dual-species biofilm model was composed of *S. mutans* UA159 and fluconazole-resistant *C. albicans*. The treatment groups consisted of Control, ZER, DNase, DEX, ZER + DNase, ZER + DEX, DNase + DEX, and ZER + DNase + DEX. Statistical comparisons among groups were performed using one-way ANOVA followed by Tukey’s post hoc test for multiple comparisons. Different letters indicate statistically significant differences between groups (*p* < 0.05). CLSM = confocal laser scanning microscopy.

For resistant biofilms (Figure 6C and 6D), the highest reduction in live/dead cells was observed with DNase alone and DNase + DEX (∼45%/55%), followed by ZER + DNase, ZER + DEX, and ZER + DNase + DEX (∼50%/50%). Furthermore, the results observed with DNase alone and DNase + DEX were significantly different from those of ZER + DNase, ZER + DEX, and ZER + DNase + DEX (*p* < 0.01).

## Discussion

In the context of oral health, matrix-degrading enzymes, in particular DNase and Dextranase, have been considered as potential therapeutic adjuncts for biofilm control and for prevention of oral diseases of microbial origin. In previous studies, DNase presented antibiofilm activities against *S. mutans* and *C. albicans* biofilms in vitro and in vivo studies.^18,26,27^ In addition, it has been reported that ZER can reduce extracellular matrix components, including polysaccharides, extracellular DNA, and proteins.^18^ However, these matrix components were not directly quantified in the present study, as they served as mechanistic background for the rationale of the proposed approach. Therefore, this study evaluated the effects of ZER alone and in combination with DNase and Dextranase on the viability and structural organization of mixed-species biofilms composed of fluconazole-susceptible and fluconazole-resistant *C. albicans* and *S. mutans*. The experimental design focused on treatment-group comparisons within a standardized biofilm model rather than benchmarking against clinically used antimicrobial agents.

Overall, the most significant reduction in colony counts for both biofilms evaluated was found in the inhibition assay. Treatment with ZER alone produced effects on biofilm formation similar to those observed with the combined treatments DNase + DEX and ZER + DEX against fluconazole-susceptible *C. albicans* and *S. mutans* biofilms. Our results align with previous research demonstrating that ZER significantly impairs *C. albicans* adhesion to surfaces and reduces its biofilm metabolic activity by up to 83.5%.^17^ In a previous study, an 8-h pretreatment of acrylic resin surfaces with ZER at 256 μg/mL led to a marked reduction in cell viability by impairing microbial adhesion and consequently inhibiting biofilm development.^19^ Similarly, ZER (128 μg/mL) was shown to markedly decrease the metabolic activity of *C. albicans* and *Staphylococcus aureus* in mixed-species biofilms by as much as 98.9%.^17^ ZER, a monocyclic sesquiterpene, is known to alter membrane permeability and fluidity, which may interfere with fungal adhesion processes.^28^ Additionally, these compounds suppress the yeast-to-hyphae transition, a critical step in biofilm maturation, thereby disrupting biofilm formation.^29–31^ Collectively, these findings, including ours, support the antibiofilm potential of ZER, which appears to act by inhibiting microbial adhesion, reducing metabolic activity, and ultimately decreasing cell viability.

On the other hand, in mixed biofilm containing fluconazole-resistant *C. albicans* and *S. mutans* a significant reduction in colony count in biofilm formation was achieved only by the combination of ZER + DEX. Moreover, ZER + DEX significantly reduced the dry weight and biomass of both types of biofilms. The observed reduction in cell viability during biofilm formation after treatment is consistent with the 2024 findings of Rikvold et al.,^27^ who investigated the antimicrobial effects of mutanase, DNase, and β-glucanase on biofilm prevention in a saliva-derived biofilm model dominated by *Streptococcus* species. The authors suggested that the decrease in biofilm viability may result from enzymatic disruption of key extracellular matrix components, which weakens the biofilm structure and enhances its removal during mechanical shear applied in subsequent washing steps.

Although theoretical support suggests that Dextranase could act on the dextran-rich polysaccharide matrix primarily produced by *S. mutans*, no studies to date have evaluated its use in degrading mixed-species biofilms of *C. albicans* and *S. mutans*. The significant effects of the ZER + DEX combination treatment on the prevention and removal of both biofilms evaluated suggest that the matrix harbored polysaccharides comprising α-glucosidic linkages, which have been less evaluated than other enzymes in dental biofilm research;^32^ however, polysaccharide composition was not directly characterized in the present study. The proposed mechanism of action involves destabilizing the extracellular matrix by targeting dextran, a key structural component synthesized by *S. mutans*.^33^ It was observed that the glucans synthesized on the *C. albicans* cell surface were more susceptible to Dextranase than those formed on salivary pellicle-coated hydroxyapatite (sHA) or the *S. mutans* cell surface. This heightened susceptibility suggests a higher proportion of α-1,6-linked glucose residues within the polymer structure.^34^

In biofilm disruption analysis, the combination of ZER + DNase showed promising antimicrobial activity against mixed-species biofilms, including fluconazole-resistant *C. albicans* + *S. mutans* biofilms. This effect was observed for the total microbiota (2.43 and 3.04 log_10_ reduction vs control group for CaS + Sm and CaR + Sm, respectively) and for specific medium for counting of *C. albicans* (2.08 log_10_ and 1.23 log_10_ reduction vs control group for CaS and CaR, respectively) and *S. mutans* (2.58 and 1.96 and log_10_ reduction vs control group, in a susceptible and resistant biofilm, respectively). In 2019, Panariello et al.^12^ observed that DNase alone did not significantly reduce the viability of fluconazole-susceptible *C. albicans*; however, they reported a significant decrease in extracellular matrix eDNA levels in the DNase-treated group compared to the untreated control. This finding underscores the role of eDNA as a key structural component of the ECM. Since eDNA contributes to biofilm stability and resistance to antifungal agents, its enzymatic degradation likely enhanced the antibiofilm activity of ZER. These results support the rationale that targeting eDNA can destabilize the ECM, facilitating increased susceptibility of microbial cells to ZER, particularly in complex, mixed-species biofilms.

Our results demonstrated a significant reduction in dry weight of biofilm after treatment with ZER, on the other hand, in a previous study, no alterations in the dry weight of *C. albicans* monospecies biofilm were observed after treatment with ZER at the same concentration.^18,35^ These results suggest that ZER treatment can be more effective against α-glucans, which are more predominant in mixed biofilm of *C. albicans* and *S. mutans* than in *C. albicans* monospecies biofilms, where ß-glucan are more abundant.^36^ Thus, by damaging α-glucans structures, ZER can directly act on the microbial surface leading to a reduction in microbial viability in both types of mixed biofilm evaluated. The findings of the present study are consistent with those of our previous work, which investigated the antimicrobial effects of ZER on mixed biofilms of *C. albicans* and *S. mutans* formed on acrylic resin surfaces.^19^ In that study, a more pronounced reduction in viable cell counts was observed, contrasting with the results reported by Abreu-Pereira et al. (2023),^18^ who evaluated monospecies biofilms of *C. albicans*. These findings, in agreement with our current data, are consistent with previous reports suggesting that ZER may exhibit enhanced activity against α-glucans, which are key structural components of biofilms involving *S. mutans*. However, as α-glucans were not directly evaluated in the present study, this mechanism requires further investigation.

Regarding the evaluation of the total biomass (dry weight) after evaluation of biofilm inhibition and after disruption of mixed biofilm of *S. mutans* and fluconazole-susceptible and -resistant *C. albicans*, ZER associated with enzymes suggested a potential synergistic effect. The ZER + DEX group showed the best results in reducing dry weight in all experimental situations. In the context of 48-h biofilm biomass, our results showed 34% and 50% reduction of the susceptible and resistant mixed biofilm, respectively, after ZER + DEX treatment when compared to the control. These results are according to others in the literature that achieved a 13% reduction of the biomass in monospecies *C. albicans* biofilm and 15% in mixed resistant biofilm (CaR + Sm) after ZER treatment alone.^18,19^

Biomass was also evaluated by crystal violet assay. This indirect technique is based on the ability of the cationic dye crystal violet to bind to negatively charged components of the biofilm, including the surfaces of live and dead microbial cells and the extracellular matrix (eDNA, polysaccharides, proteins).^37^ On the other hand, dry-weight analysis technique involves physically separating the biofilm from the culture medium, drying it thoroughly to remove all water content, and then weighing the remaining material. It provides a direct measure of the total dry weight of the biofilm, encompassing all cellular and extracellular components after water has been removed.^38^ The differences between these procedures were likely responsible for the differences found in our results, in which ZER was able to reduce a significant amount of biomass in the crystal violet assay in both biofilms evaluated, and this significant reduction was not observed in dry-weight analysis.

Resistance to mechanical clearance represents another important mechanism by which biofilms persistently attach to abiotic and biotic surfaces, making physical removal challenging.^39,40^ CLSM images and cell quantification also demonstrate the ability of ZER and EPS-degrading enzymes to induce microbial cell dispersal and cause structural collapse of the biofilm architecture, indicating disruption of the biofilm scaffold. A similar result was found in a previous study that also evaluated the effect of ZER on biofilms of fluconazole-resistant *C. albicans* clinical isolates.^35^ Dispersed cells from biofilms are more sensitive to killing by antimicrobial agents.^20^ However, dispersed cells can restart the biofilm life cycle,^41^ reinforcing the concept that EPS-targeting approaches should be coadministered with antimicrobials. Thus, the combination of EPS degradation and ZER has potential as an antibiofilm therapeutic approach.

Our results varied according to the *C. albicans* strain used, with distinct responses observed between biofilms formed by fluconazole-susceptible and fluconazole-resistant strains. Biofilms formed by fluconazole-resistant *C. albicans* in association with *S. mutans* may differ from those formed by fluconazole-susceptible strains not only in antifungal susceptibility, but also in biofilm architecture, extracellular matrix composition, and stress tolerance.^9,10,18,24,36^ In mixed biofilms, *S. mutans*-derived glucosyltransferases promote the accumulation of extracellular α-glucans on the fungal surface, enhancing coadhesion, biomass formation, and structural complexity. When this interaction occurs with a fluconazole-resistant *C. albicans* strain, additional resistance-associated mechanisms, including altered membrane biology, efflux pump activity, stress adaptation, and matrix-mediated protection, may further reduce treatment susceptibility.^9,10,18,24,36^ Therefore, differences between susceptible and resistant mixed biofilms may reflect both strain-dependent fungal phenotypes and cross-kingdom interactions with *S. mutans*, which together influence biofilm biomass, matrix organization, and antimicrobial tolerance. In contrast, fluconazole resistance should not be interpreted as a general indicator of reduced susceptibility to all antibiofilm treatments, since resistance mechanisms may be drug- or target-specific.^42–44^ Therefore, fluconazole-resistant *C. albicans* biofilms may exhibit distinct responses to nonazole strategies that affect extracellular matrix organization, biofilm architecture, or microbial viability through mechanisms unrelated to ergosterol biosynthesis.^42–44^

Although ZER, DEX, and DNase may theoretically interact within the biofilm microenvironment, they are unlikely to share the same binding sites because their primary molecular targets are distinct.^18,45,46^ Still, antagonism would not be expected to result from competition for the same biofilm-binding sites, but rather from possible physicochemical incompatibility or interference with enzymatic activity. Moreover, DNase I requires divalent cations, such as Mg^2+^ and Ca^2+^, to maintain adequate enzymatic activity.^47^ Hence, formulation components, buffers, chelating agents, solvents, or pH changes may impair its activity independently of the presence of DEX. This may represent a possible reason that, in some experimental assays, the combined treatments did not produce statistically robust effects, particularly when ZER, DEX, and DNase were administered concomitantly.

Although the present in vitro study provides controlled and reproducible evidence of the antibiofilm efficacy of the tested treatments, the experimental nature of this model inherently restricts direct extrapolation to clinical settings. For example, cytotoxicity assays were not performed in the present study; however, the biological safety profile of the evaluated treatments has previously been assessed under comparable experimental conditions.^35,48–50^ While the antibiofilm effects were demonstrated through viability- and biomass-based assays, additional analytical approaches are required to directly elucidate potential changes in the composition and spatial organization of the extracellular matrix. Therefore, the proposed mechanisms involving matrix degradation and disruption in the present study should be interpreted as hypothesis-generating and based on previous literature rather than direct experimental evidence.

In addition, the confocal microscopy analysis performed in this study was limited to live/dead staining and therefore did not allow direct visualization of extracellular matrix components. Future investigations should employ EPS-specific fluorescent probes, such as lectin-based polysaccharide staining and eDNA-targeting dyes, to better characterize treatment-induced changes in matrix architecture and composition.

Further investigations should incorporate biochemical assays targeting eDNA, proteins, and extracellular polysaccharides, as well as advanced imaging approaches to evaluate biofilm architecture, thickness, and matrix organization. Moreover, investigations should incorporate cytotoxicity assays, checkerboard assays to further characterize potential synergistic interactions between the compounds, concentration–response assays to determine Minimum Biofilm Inhibitory Concentration (MBIC) and Minimum Biofilm Eradication Concentration (MBEC) values to further characterize the dose-dependent interactions and efficacy of these compounds, comparisons with standard antimicrobial treatments, and complementary imaging-based analyses. These studies should be performed both in vitro and in appropriate animal models to further validate the mechanisms involved and strengthen the translational relevance of the proposed therapeutic approach.

## Conclusion

In conclusion, the present study shows that ZER exerts significant antibiofilm activity against mixed-species biofilms of *C. albicans* and *S. mutans*, and that its combination with ECM-degrading enzymes further enhances biofilm disruption. Importantly, ZER alone also demonstrated marked activity, highlighting its intrinsic antibiofilm potential. The standardized mixed-species biofilm model enabled a reproducible assessment of treatment effects and provides a foundation for future investigations in more complex, clinically relevant biofilm systems. Overall, these findings support the use of ZER alone or in combination with ECM-degrading enzymes as a potential therapeutic strategy for oral microbial infections.
